# Implementation of GOLD consensus report in real life: results from the Velletri-Lariano (VELA) cohort

**DOI:** 10.1186/s40248-017-0095-2

**Published:** 2017-07-17

**Authors:** G. De Filippi, M. Lallini, G. De Riggi, G. Marchetti, C. M. Dimartino, A. M. Russetti, E. Ferrari, R. Pistelli, M. S. Magnoni, M. Riparbelli, A. Rizzi, P. Angeletti

**Affiliations:** 1 Ospedale Paolo Colombo, Velletri (RM), Italy; 2 Azienda Roma H, M M G Dist. H5 ASL RmH, Rome, Italy; 3 Università Cattolica Del Sacro Cuore - Complesso Associato Columbus, Rome, Italy; 4 GSK Medical Department, Verona, Italy

**Keywords:** COPD, Exacerbations, Co-morbidities, CAT, GOLD guidelines

## Abstract

**Background:**

COPD is one of the leading causes of morbidity and mortality. Pharmacotherapy improves quality of life and reduces exacerbations although low adherence with prescribed treatments may represent a barrier to optimal disease management.

The first objective of this paper is to report the distribution of COPD patients according to GOLD categories, in a sample of patients from a cohort study in an area of the Latium region in Italy. The second objective is to evaluate the agreement between the distributions of severity obtained from the HCPs and the experts included in the study board (Board).

**Methods:**

COPD patients were given a card to collect demographic and clinical data at baseline. Information in those cards was independently evaluated by HCPs and Board to include each patient into one of the four GOLD categories.

**Results:**

In a sample of 187 stable COPD patients, 59% male, mean age 70 year, the distribution of GOLD categories according to the Board was: 6% A, 34% B, 2% C, and 58% D. A discrepancy in GOLD classification was observed between the study board and field-based HCPs, regarding more than 50% of the patients, with a clear trend to underestimate the frequency of patients in D level (21%) and to overestimate the frequency in C level (21%).

**Conclusions:**

These results describe for the first time the distribution of COPD patients in an Italian cohort according to the GOLD categories, with the highest frequencies in levels B and D. The misclassification from HCPs may impact the therapeutic approach and the clinical outcomes.

## Background

The increasing prevalence of chronic illness is posing considerable challenges to health systems. Because of the increasing life span of general population and the persisting smoking habit, chronic obstructive pulmonary disease (COPD) is expected to become the third cause of death worldwide by 2020. Currently, in Italy the prevalence of COPD is estimated around 4.5% of the general population [1], in line with the international data [2, 3].

Among respiratory diseases, COPD is one of the leading causes of morbidity and mortality worldwide, with a high economic burden due to hospital admissions or emergency department (ED) visits for exacerbations or complications of the disease. An Italian observational study reported that the mean annual cost per patient is estimated around 3723 Euro and that hospitalization and emergency visits for exacerbations are the major cost driver of COPD, corresponding to about 65% of direct costs of illness. Altogether, COPD accounts for about 6% of the Italian health expenditure [4]. Pharmacotherapy has been shown to significantly improve quality of life, reduce exacerbations and potentially the risk of death [5, 6], although adherence with prescribed treatments is frequently poor in COPD [7–9], representing a significant barrier to optimal management, with both clinical and economic consequences.

These considerations raise the importance of timely diagnosis, correct assessment, and appropriate treatment of COPD patients. There are a number of national and international strategy documents and guidelines aiming at appropriateness of COPD diagnosis and therapy [10–13]. Since GOLD report is an international consensus on COPD management, the objective of the present study was to examine how correctly healthcare professionals (HCP) (from Local Health Organization and Primary Care in ROMA 5H district) were applying patient classification according to multidimensional GOLD categories in a cohort of COPD patients (Velletri-Lariano, VELA cohort).

## Methods

**Fig. 1:**
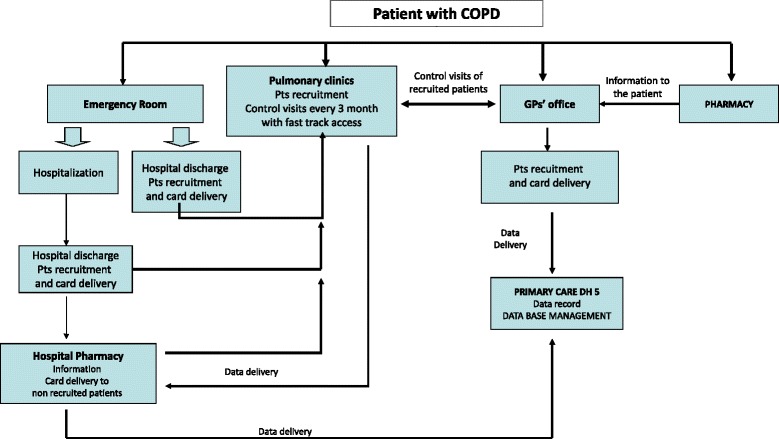
Flow chart of the pathways for patient recruitment

Participants were recruited through various mechanisms, including general public advertising (at schools, nursing homes, community pharmacies, etc.) and screening in primary-care and pulmonary clinics. All subjects were given a card to obtain a preferential access to pulmonary clinics for lung function test. Patients were eligible if they had a diagnosis of COPD according to GOLD consensus report, on the basis of symptoms, clinical history, and post-bronchodilator FEV_1_/FVC < 0.70. The flow chart reported in Fig. 1 describes the different pathways of patient recruitment and follow up.

The project and the analysis of the data were conducted with approval from the relevant local authority (ROMA 5H district).

All participants signed the informed consent form and were instructed to correctly fill into the card all the information required, with a peculiar attention to CAT score, and number of episodes of exacerbation in the last year (defined by use of antibiotics and/or systemic steroids or admission to the hospital related to worsening of respiratory symptoms).

The following patient’s demographic and clinical data were reported on the card at baseline: age, gender, co-morbidities (diabetes, hypertension, atrial fibrillation, heart failure, ischemic cardiac disease, dislipidemia), smoking history, exacerbations during the previous year, quality of life according the Italian version of the CAT (scale 0–40), current treatments, and lung function.

Data were anonymized in a database with hierarchical access control in order to guarantee secure information access. The observational period was from May 7^th^ 2013 to April 29^th^, 2015.

Data were analysed in order to describe the characteristics of patients affected and treated for COPD in a real clinical practice. Patient’s were classified according to GOLD A, B, C and D categories (2013 update) by the study board and by HCPs participating in the program, and the two distributions were compared. GOLD categories are based on a multidimensional assessment method based on FEV_1_, exacerbation history, and symptom scores (measured using either the modified Medical Research Council [mMRC] or the COPD Assessment Test [CAT] score).

In order to verify whether, in the course of time, the agreement between the study board and HCPs in patient classification had improved, we repeated the analysis by splitting the data collected in the first and the second half of the recruitment period.

### Statistical analysis

We used only descriptive statistical analysis. We measured the mean number of exacerbations in the last 12 months, mean FEV_1_, SaO_2_, and CAT score, GOLD classification, frequency of different co-morbidities, frequency of risk factors for COPD, frequency of long term oxygen therapy and of any pharmacological treatmentPatient classifications according to the board and the HCPs were compared in two-way tables.

## Results

Three hundred and twenty nine patients were observed. For the sake of this survey, data from 142 patients were not analyzed because one or more mandatory information for GOLD classification were missing (129 had missing CAT scores). The main characteristics of the remaining 187 patients are reported in Table 1.

**Table 1:** Patients baseline characteristics

- N. Patients	187
- Mean age (yrs)	70.9 ± 1
- Smoking history	22% ex smokers
	18% non smokers
	60% current smokers
- CAT score	20 ± 7
- Patients with cardiovascular co-morbidities^a^	65%
- Patients in oxygen therapy	18.1%
- Patients with COPD-related hospitalizations in the past year	21.3%
- Patients treated with respiratory medications in the last year	88%

^**a**^Diabetes, hypertension, heart failure, ischemic coronary artery disease, atrial fibrillation, dislipidemia

**Fig. 2:**
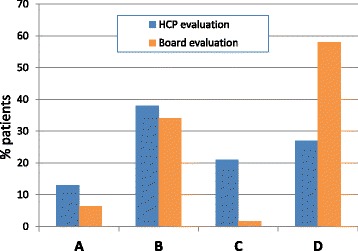
GOLD (update 2013) classification of COPD patients according to the study board and HCPs

On the basis of CAT, FEV_1_ and exacerbation history, the distribution of patient’s categories according to the study board was: 6% in level A, 34% in level B, 2% in level C, and 58% in level D (Fig. 2). The frequency of patients affected by cardiovascular co-morbidities in the different levels of severity of COPD was: 42% (5/12) in level A, 55% (35/63) in level B, 100% (3/3) in level C, and 72% (79/109) in level D.

According to HCPs, the distribution of patients was: 14% in level A, 38% in level B, 21% in level C, and 27% in level D (Fig. 2).

Overall, we found a surprisingly low level of agreement in the patient classification according to GOLD guidelines between the study board and HCPs, the latter showing a clear trend to underestimate or overestimate the prevalence of patients in level D or C, respectively (Fig. 2).

On closer examination, an agreement on COPD classification was obtained in 81 out of 187 patients (43%), whereas COPD impact was underestimated in 86 out of 187 (46%) and overestimated in 20 out of 187 (11%) patients (green, red, and light blue figures in Table 2).

**Table 2:**
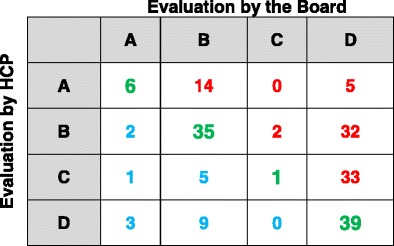
Patients classified by the board vs patients classified by HCPs

Chi square = 49.91

Degrees of freedom = 9

*p* < 0.0001

Crude agreement = (6 + 35 + 1 + 39)/187*100 = 43%

Green = concordant classification; Red = underestimation of staging by HCP’s; sky-blue = overestimation of staging by HCP’s

By comparing the level of agreement achieved during the first and the second half of the recruitment period, we didn’t find any improvement in the agreement of severity scoring (Tables 3 and 4).

**Table 3:**
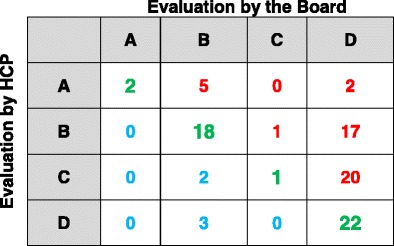
Patients classified by the board vs patients classified by the first group of HCPs enrolled before 31.03.2014

Chi square = 40.82

Degrees of freedom = 9

*p* < 0.0001

Crude agreement = (2 + 18 + 1 + 22)/93*100 = 46%

Green = concordant classification; Red = underestimation of staging by HCP’s; sky-blue = overestimation of staging by HCP’s

**Table 4:**
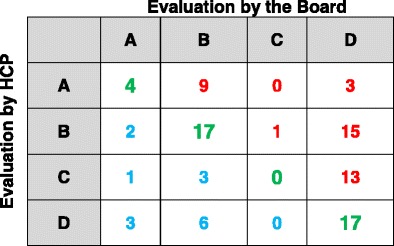
Patients classified by the board vs patients classified by the second group of HCPs enrolled after 31.03.2014

Chi square = 18.81

Degrees of freedom = 9

*p* = 0.026

Crude agreement = (4-17 + 0 + 17)/94*100 = 40%

Green = concordant classification; Red = underestimation of staging by HCP’s; sky-blue = overestimation of staging by HCP’s

## Discussion

The data describe the characteristics of patients affected by COPD and the staging of their disease according to the GOLD classification in a clinical practice reality in this country.

The mean age of our patients was higher than the age of patients studied in the most important clinical trials on COPD [14–16]. The frequency of comorbidities was also very high as well as the frequency of subjects on long term oxygen therapy, both important criteria for the exclusion of those subjects from any clinical trial. The highest prevalence of patients was found in level B (34%) and D (58%), in line with results from literature [17, 18]. In keeping with data from several epidemiological studies [19], concomitant cardiovascular diseases were found in a large proportion of subjects of our cohort and were mainly reported in more symptomatic patients (CAT ≥ 10) corresponding to the B and D levels of GOLD categories. Since patients were recruited from pulmonary clinics, general medical practice and also general community advertising, this cohort may represent a realistic distribution of COPD impact/clinical risk in the general population.

The discrepancy in GOLD classification between the study board and field-based HCPs involved in this survey, regarding more than 50% of the patients, is a novel and unexpected finding. An important driver responsible for GOLD misclassification seems to be the underestimation of disease impact/clinical risk, mainly due to an incorrect evaluation of symptoms, with an underestimation by HCPs of the group D (high level of risk and high level of symptoms) and an overestimation of the group C (high level of risk but low level of symptoms). These results possibly reflect a discrepancy between patient’s perception and clinician’s assessment of symptoms or an underestimation by HCPs of CAT as a tool able to measure the impact of COPD symptoms on patient’s life.

Interestingly, a survey conducted in the Czech Republic using 1355 patient records showed a 32.8% misclassification by respiratory specialists, as compared with an objectively computed categorization, that was mainly based on errors in the assessment of symptoms. The misclassification resulted in 15.4% of patients receiving ICS unnecessarily, whereas in 15.8% patients ICS were erroneously omitted [20].

However, also an underestimation of the clinical risk by HCPs was observed. Lastly, a small but not negligible overestimation of COPD risk was also observed.

Notably, during the first and the second half of the survey period no improvement was observed in the agreement of classification between HCPs and the study board. The reasons of this finding are not clear and need further investigation.

Co-morbidities represent an independent factor of mortality and hospitalization in subjects with respiratory impairment and should be actively looked for and appropriately treated [5]. Unfortunately, the results of this study confirm that the majority of subjects affected and treated for COPD in the clinical practice would never be enrolled in clinical trials, so that translating the results of these trials to the real world is just a matter of clinical art and not of scientific evidence.

This pilot study has several limitations: the sample population is small and HCP are from a specific geographic area, thus the observed data may not reflect the general clinical practice in Italy. Another limitation, frequently reported in observational studies, is that there were several missing data in the patient card, thus limiting the sample of patients that could be analyzed.

## Conclusions

Our data clearly indicate that real-life implementation of GOLD strategy, as regards patients’ ABCD categorization, is poor, with an underestimation of symptom impact being the main driver of the erroneous classification. This finding could negatively affect the therapeutic approach and clinical outcomes. An improved awareness of CAT as a validated, simple, reliable instrument to measure the overall COPD-related health status and impact on individual patients is urgently needed.

## Abbreviations

CAT: COPD assessment test
FEV_1_: Forced expiratory volume in the first second
GOLD: Global Initiative for Chronic Obstructive Lung Disease
HCP: Health care provider
mMRC: Modified Medical Research Council
SaO_2_: Oxygen saturation
VELA: VElletri-LAriano
